# Supplementary material for: Causal Relationship between Aging and Anorexia Nervosa: A White-Matter-Microstructure-Mediated Mendelian Randomization Analysis
Source: Biomedicines. 2024 Aug 16;12(8):1874. doi: 10.3390/biomedicines12081874 (PMC11351342; doi:10.3390/biomedicines12081874)
Supplement: Supplementary file 1 [file biomedicines-12-01874-s001.zip › Supplementary Figure/Figure S4/Singe SNP plot of MR analysis for TL to ubm-b-1940.pdf]

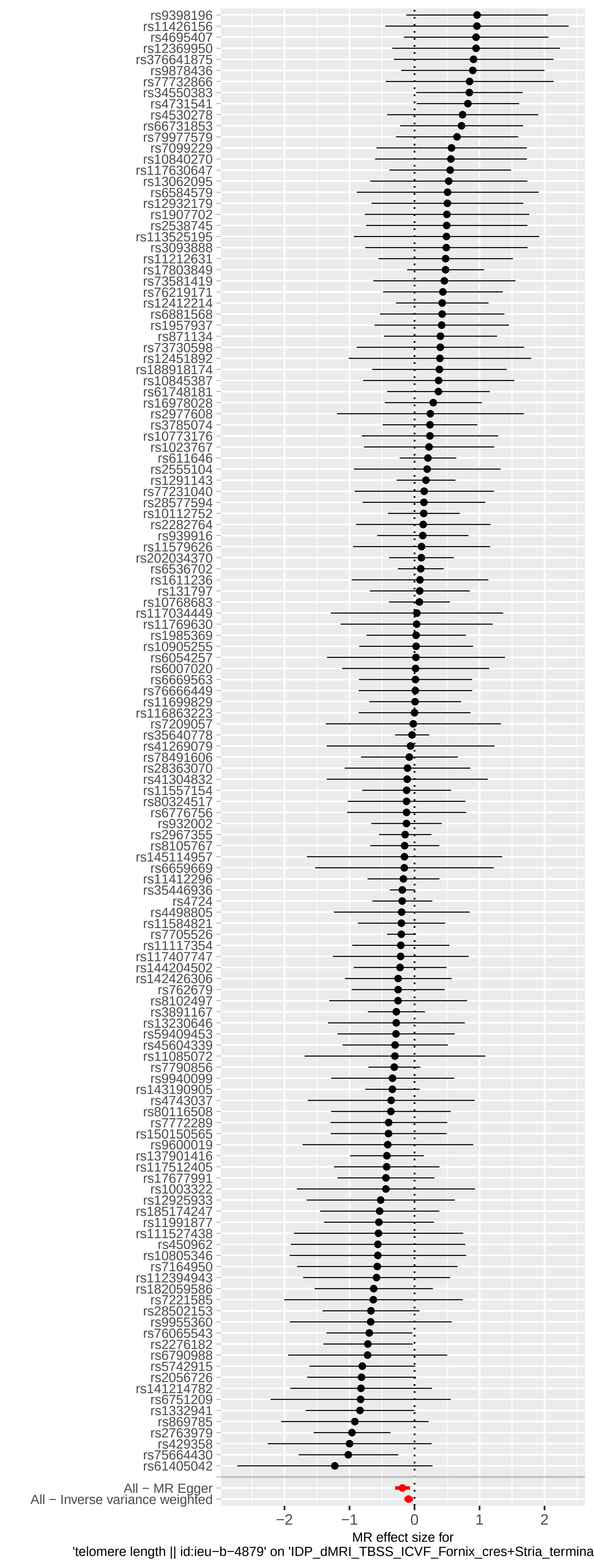

rs9398196
rs11426156
rs4695407
rs12369950
rs376641875
rs9878436
rs77732866
rs34550383
rs4731541
rs4530278
rs66731853
rs79977579
rs7099229
rs10840270
rs117630647
rs13062095
rs6584579
rs12932179
rs1907702
rs2538745
rs113525195
rs3093888
rs11212631
rs17803849
rs73581419
rs76219171
rs12412214
rs6881568
rs1957937
rs871134
rs73730598
rs12451892
rs188918174
rs10845387
rs61748181
rs16978028
rs2977608
rs3785074
rs10773176
rs1023767
rs611646
rs2555104
rs1291143
rs77231040
rs28577594
rs10112752
rs2282764
rs939916
rs11579626
rs202034370
rs6536702
rs1611236
rs131797
rs10768683
rs117034449
rs11769630
rs1985369
rs10905255
rs6054257
rs6007020
rs6669563
rs76666449
rs11699829
rs116863223
rs7209057
rs35640778
rs41269079
rs78491606
rs28363070
rs41304832
rs11557154
rs80324517
rs6776756
rs932002
rs2967355
rs8105767
rs145114957
rs6659669
rs11412296
rs35446936
rs4724
rs4498805
rs11584821
rs7705526
rs11117354
rs117407747
rs144204502
rs142426306
rs762679
rs8102497
rs3891167
rs13230646
rs59409453
rs45604339
rs11085072
rs7790856
rs9940099
rs143190905
rs4743037
rs80116508
rs7772289
rs150150565
rs9600019
rs137901416
rs117512405
rs17677991
rs1003322
rs12925933
rs185174247
rs11991877
rs111527438
rs450962
rs10805346
rs7164950
rs112394943
rs182059586
rs7221585
rs28502153
rs9955360
rs76065543
rs2276182
rs6790988
rs5742915
rs2056726
rs141214782
rs6751209
rs1332941
rs869785
rs2763979
rs429358
rs75664430
rs61405042
All − MR Egger
All − Inverse variance weighted
−2
−1
0
1
2
MR effect size for
'telomere length || id:ieu−b−4879' on 'IDP_dMRI_TBSS_ICVF_Fornix_cres+Stria_termina
